# *Bifidobacterium bifidum* CIP-01 attenuates metabolic dysfunction-associated steatotic liver disease induced by high-alcohol-producing *Klebsiella pneumoniae*

**DOI:** 10.1007/s00535-025-02332-x

**Published:** 2025-12-24

**Authors:** Xue Ren, Chao Yan, Xuanfeng Liu, Xinyu Jia, Yujie Chen, Hanqing Zhao, Yanling Feng, Guanhua Xue, Jinghua Cui, Yuehua Ke, Lin Gan, Junxia Feng, Zheng Fan, Tongtong Fu, Ziying Xu, Zihui Yu, Yang Yang, Rentao Yu, Jing Yuan

**Affiliations:** 1 Capital Institute of Pediatrics, Chinese Academy of Medical Sciences & Peking Union Medical College, Beijing, China; 2 Capital Center for Children’s Health, Capital Medical University, Capital Institute of Pediatrics, Beijing, China; 3 Grand Life Sciences Group Co., Ltd, China Beijing,

**Keywords:** High-alcohol-producing *Klebsiella pneumoniae*, MASLD, *Bifidobacterium bifidum*, Probiotic therapy

## Abstract

**Background:**

High-alcohol-producing *Klebsiella pneumoniae* (HiAlc *Kpn*) can cause metabolic dysfunction-associated steatotic liver disease (MASLD) through sustained alcohol overflow in the microenvironment. As a probiotic, *Bifidobacterium bifidum* (*B. bifidum*) exhibits unique anti-inflammatory properties; however, whether and how it alleviate MASLD induced by HiAlc *Kpn* requires further investigation.

**Methods:**

MASLD mouse model was constructed by gavage administration with HiAlc *Kpn* W14 to assess the therapeutic effect of *B. bifidum* CIP-01 in vivo. Cell infection models, metabolomics sequencing, and in vitro antibacterial assays were integrated to systematically elucidate the mechanism by which *B. bifidum* CIP-01 mitigates HiAlc *Kpn* W14-induced cell damage.

**Results:**

*B. bifidum* CIP-01 was able to ameliorate MASLD induced by HiAlc *Kpn* through a multi-target mechanism. Compared to pair-fed mice, HiAlc *Kpn* W14 disrupted gut barrier and promoted inflammatory cytokines release. While, supplementation with *B. bifidum* CIP-01 reversed these effects by a) restoring intestinal integrity via upregulating tight junction proteins (ZO-1/Occludin) and mucin protein MUC-2, reducing reactive oxidative stress (ROS) and apoptosis in colonic cells, and b) rescuing hepatic cytochrome P450 2E1 (CYP2E1)-driven oxidative injury (ROS/Caspase-3) while promoting mitochondrial *β*-oxidation, as well as c) directly suppressing HiAlc *Kpn* proliferation and biofilm formation. Metabolomics and 16S rRNA of fecal samples analyses revealed *B. bifidum* CIP-01-mediated metabolic regulation: depletion of toxic branched-chain amino acids (BCAAs) intermediates and restoration of energy homeostasis and antioxidant defense alongside increased short-chain fatty acids (SCFAs)-associated pathways.

**Conclusion:**

Our findings highlight *B. bifidum* CIP-01 as a novel therapeutic candidate for HiAlc *Kpn*-induced MASLD, operating through a triad of pathogen suppression, gut–liver axis repair, and metabolic regulation.

**Supplementary Information:**

The online version contains supplementary material available at https://doi.org/10.1007/s00535-025-02332-x.

## Introduction

Metabolic dysfunction-associated steatotic liver disease (MASLD) is a common chronic liver disease worldwide, with a complex and multifactorial etiology. Insulin resistance [1], genetic predisposition [2], oxidative stress, and endoplasmic reticulum stress [3] contribute to its onset and progression. In addition, dysregulation of the intestinal microbiota compromises gut barrier function in the upper cortex by increasing permeability, decreasing intercellular junction integrity, and facilitating the translocation of pathogens and their metabolites to the liver, thus triggering inflammation, immune responses, and hepatic injury [4–6].

Notably, intestinal colonization by certain pathogens, such as *Klebsiella pneumoniae* (*K. pneumoniae*), may promote the development of hepatocellular carcinoma [7]. In patients with primary sclerosing cholangitis, *K. pneumoniae* has been found to disrupt the epithelial barrier, leading to bacterial translocation and hepatic inflammation [8]. Our previous study demonstrated that—even in healthy individuals—*K. pneumoniae* can impair intestinal barrier function by producing substantial quantities of endogenous ethanol, resulting in intestinal leakage and subsequent translocation of the bacteria and its metabolites to the liver, where they cause inflammation and tissue damage [9, 10].

To address the multi-organ damage associated with *K. pneumoniae*, various targeted therapeutic strategies are under investigation. Phage therapy has been shown to reduce alcohol-induced hepatic injury, inflammation, and metabolic dysfunction in MASLD [10]. DNA methylation may also influence liver injury induced by HiAlc *Kpn*, presenting potential therapeutic targets [11]. Furthermore, probiotics and specific antibodies may reduce bacterial colonization, modulate gut microbiota composition, and enhance immune responses, thus mitigating organ damage [12–15].

*Bifidobacterium bifidum* (*B. bifidum*), a well-studied probiotic, exhibits multiple therapeutic mechanisms relevant to MASLD. It can improve the intestinal microenvironment by increasing the relative abundance of beneficial bacteria while suppressing harmful strains, thus reducing hepatic inflammation and lipid accumulation [16]. Moreover, it may regulate lipid metabolism by inhibiting lipid synthesis and potentially enhancing production of SCFAs in the liver [17]. In addition, *B. bifidum* may exert therapeutic effects by enhancing gut barrier function, limiting intestinal endotoxin translocation to the liver, and attenuating hepatic inflammatory responses.

Previous studies have identified *B. bifidum* S17 as a potent antagonist of lipopolysaccharide-induced nuclear factor (NF)-*κ*B activation and interleukin-8 secretion, demonstrating strong adhesion to cultured intestinal epithelial cells [18]. The anti-inflammatory capacity of this strain has been further validated in two mouse models of colitis [19, 20]. *K. pneumoniae* W14 (HiAlc *Kpn*), an alcohol-producing gut pathobiont, has been established as a driver of alcohol-independent hepatic steatosis via sustained intestinal alcohol overflow [9]. Based on this model, we investigated the effects of probiotic treatment with *B. bifidum* CIP-01 on the following: (a) protection of gut barrier function in response to *K. pneumoniae* infection; (b) reduction of liver injury caused by HiAlc *Kpn* and its metabolites in circulation; and (c) modulation of HiAlc *Kpn* metabolism.

## Methods

### Animals and treatment with *B. bifidum* CIP-01

**Fig. 1:**
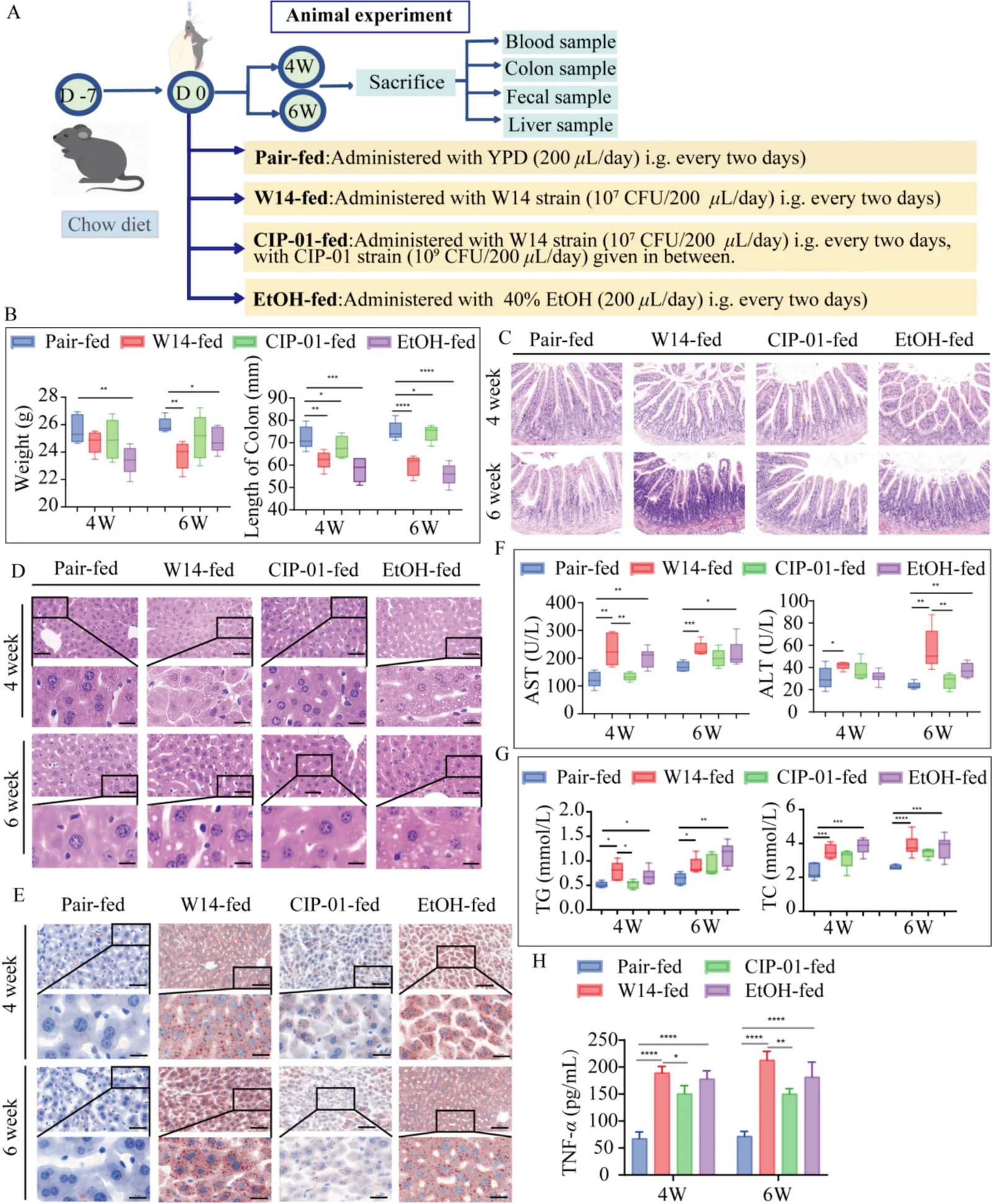
*B. bifidum* CIP-01 ameliorates MASLD induced by HiAlc *Kpn* W14 in mice. **A** Study design for animal experiment, n = 6 for each group. **B** Quantification diagram of mouse colon length and body weight. **C** and **D** H&E staining (40X) of small intestine and liver sections from SPF mice fed with HiAlc *Kpn* W14, *B. bifidum* CIP-01, EtOH and a chow diet for 4 and 6 weeks. (**E**) Oil Red O (ORO) staining (40X) of liver sections from SPF mice. **F**–**I** The serum levels of TG and TC (**F**), and the contents of AST, ALT (**G**) and TNF-*α* (**H**) from SPF mice fed with HiAlc *Kpn* W14, *B. bifidum* CIP-01, EtOH and a chow diet for 4 and 6 weeks were detected. All data are presented as the mean ± SD of three independent experiments. *ns, P* > 0.05, ^*^P < *0.05*, ^**^P < *0.01*, ^***^P < 0.005, ^****^P < *0.001*. *P* value was assessed by two-way ANOVA

The high-alcohol-producing *K. pneumoniae* W14 was cultured and administered to mice as previously described [9]. Male C57BL/6 J mice (6–7 weeks old, Charles River Corp., Beijing, China) were randomly divided into four groups and acclimatized for 2 weeks. For 4–6 weeks, mice were inoculated intragastrically with HiAlc *Kpn* W14 (10^7^ CFU/200 *μ*L) once every 2 days, with ethanol and YPD broth as controls. *B. bifidum* CIP-01 (10^9^ CFU/200 *μ*L) was administered similarly for intervention. Anaerobic cultures were used for probiotic preparation. The specific experimental protocol refers to Fig. 1A.

### Histology and physiological analysis and gene quantification in mice

At the terminal of the experiment, fasted mice were euthanized. Body weight and colon length were recorded. Feces were collected for 16S rRNA and metabolomics. Livers were processed for H&E, Oil Red O, and qPCR. Serum was analyzed for diamine oxidase (DAO), tumor necrosis factor (TNF)-*α*, alanine aminotransferase (ALT), aspartate aminotransferase (AST), triglycerides (TG), and total cholesterol (TC). Intestinal tissues underwent H&E and IHC staining. All immunohistochemistry images were quantified using ImageJ software (v1.54, NIH) with the “IHC Profiler” plugin for standardized analysis.

### 16S rRNA sequencing

For bacterial diversity analysis, 200 mg fresh mouse feces were collected and total DNA was extracted, and the V3–V4 region was amplified. Libraries were prepped (TruSeq), sequenced (MiSeq), and analyzed via QIIME2 (DADA2/Silva v138). Negative/positive controls and replicate samples were added to verify the reliability (similarity between samples > 85%).

### Metabolomic profiling of intestinal contents

For metabolomic analysis, 50 mg intestinal contents were collected and ultrasonically homogenized. The metabolites were analyzed by GC–TOF–MS. The original data were extracted and annotated by MS-DIAL software, differential metabolites were obtained from OPLS-DA and Wilcoxon test (*P* < 0.05). Quality control samples (RSD < 15%) were inserted in each batch to ensure data stability.

### Cell culture and construction of HiAlc *Kpn*-induced cell injury model

NCM460 cells and HepG2 cells were cultured in DMEM (10% FBS, 100 U/mL penicillin and 100 mg/mL streptomycin) in a incubator with 5% CO_2_ at 37 °C. For HiAlc *Kpn* infection, cells were seeded at a density of 1 × 10^5^ cells/well overnight. After 24 h of exposure to HiAlc *Kpn* W14 (MOI = 10) alone, co-incubated with W14 strains and supernatant of *B. bifidum* CIP-01 culture medium (50 *μ*L), as well as EtOH (500 μM), respectively, for 24 h. Supernatants were analyzed for lactate dehydrogenase, IL-1*β* and TNF-*α*. Cells were harvested for protein assays.

### Cell viability assay

3 × 10^4^ cells/well (100 *μ*L medium/well) were seeded, after 24 h of HiAlc *Kpn* infection, 10 *μ*L CCK-8 solution was added and absorbance was read at 450 nm. Cell viability was calculated by (experimental group absorbance value/control group absorbance value) × 100%.

### Cytokine analysis by ELISA

Mouse serum and cell culture supernatants were collected to measure the release of TNF-*α* (E-EL-H0109/E-EL-M3063, Elabscience®), IL-1*β* (E-EL-H0253, Elabscience®) with ELISA kits according to the manufacturer’s instructions, and the absorbance was then measured at 450 nm using a microplate reader.

### Immunofluorescence microscopy analysis

After treatment, cells were stained with Hoechst33342 (nucleus), JC-1 (mitochondria), ER-Tracker (ER), CellROX (ROS), and Fluo-4 (Ca^2+^). Apoptosis was assessed using Annexin V-PE. Fluorescence was imaged and quantified.

### Western blot

HepG2 protein extracts were resolved by SDS-PAGE, transferred to PVDF membranes, and probed with anti-CYP2E1 (Proteintech) and HRP-secondary Ab. Bands were imaged and quantified using ImageJ.

### Growth curve and biofilm formation determination

The high-alcohol-producing *K. pneumoniae* W14 was cultured and diluted with YPD medium at a ratio of 1:100 and measured at 600 nm every 1 h to create the growth curve. Moreover, W14 was diluted (1:50) and cultivated in six-well plates for 24 and 48 h after culturing to identify its biofilm formation. For antibacterial assay, *B. bifidum* CIP-01 supernatant (20% v/v) was added and the pH was adjusted to 7.0 ± 0.5 with sterile NaOH/HCl prior to co-culture with W14.

### Statistical analysis

For assessing differences between the two groups, a two-tailed *t* test was performed. For differences among more than two groups, a one-way analysis variance (ANOVA) was performed. All data are shown as mean ± SD. Statistical analyses were performed with GraphPad Prism (version 10.0). Statistical significance was defined as *P* < 0.05. Results from representative experiments (such as micrographs) were obtained from at least three independent fields of view with similar results.

## Results

### *B. bifidum* CIP-01 ameliorates MASLD induced by HiAlc *Kpn* W14

After 4 and 6 weeks of administration of HiAlc *Kpn* W14 and *B. bifidum* CIP-01 (Fig. 1A), significant differences were observed in the histopathology of liver and intestinal tissues. In the W14-fed group, mice exhibited weight loss and shortened colonic length. However, supplementation with *B. bifidum* CIP-01 reversed these effects (Fig. 1B). Histological examination revealed prominent eosinophilic alterations, villous blunting, and a substantial reduction in the number of goblet cells secreting mucus in W14-fed mice (Fig. 1C). In addition, pathological assessment of liver tissue showed that hepatocytes underwent severe ballooning degeneration that involved nuclear fragmentation and dissolution (Fig. 1D), along with extensive inflammatory cell infiltration in the portal regions (Fig. S1). Numerous large and small lipid vacuoles also were observed within the hepatocyte cytoplasm (Fig. 1E). In addition to histopathological damage, W14 administration led to elevated serum levels of ALT and AST (Fig. 1F), increased concentrations of TG and TC (Fig. 1G), and heightened expression of the inflammatory cytokine TNF-*α* (Fig. 1H)*.*

The above damage was significantly attenuated in mice supplemented with *B. bifidum* CIP-01. Along with the restoration of colon length, intestinal villus length and goblet cell number showed partial recovery. Hepatocellular injury was also alleviated after *B. bifidum* CIP-01 supplementation. The structures of hepatic lobules and liver cells approached normal conditions, with reductions in both hepatic lipid accumulation and inflammatory infiltration. Furthermore, supplementation with CIP-01 reduced serum levels of AST, ALT, TC, TG, and TNF-*α* (Fig. 1B–H). However, Masson staining revealed no significantly detectable collagen deposition in the liver of experimental mice in any groups (Fig. S2).

In summary, these results suggest that HiAlc *Kpn* induces intestinal tissue injury and hepatic lipid deposition, contributing to hepatocellular inflammation and damage. However, intervention with *B. bifidum* CIP-01 effectively counteracts this pathological process and mitigates liver injury.

### *B. bifidum* CIP-01 alleviates intestinal damage induced by HiAlc *Kpn* W14

**Fig. 2:**
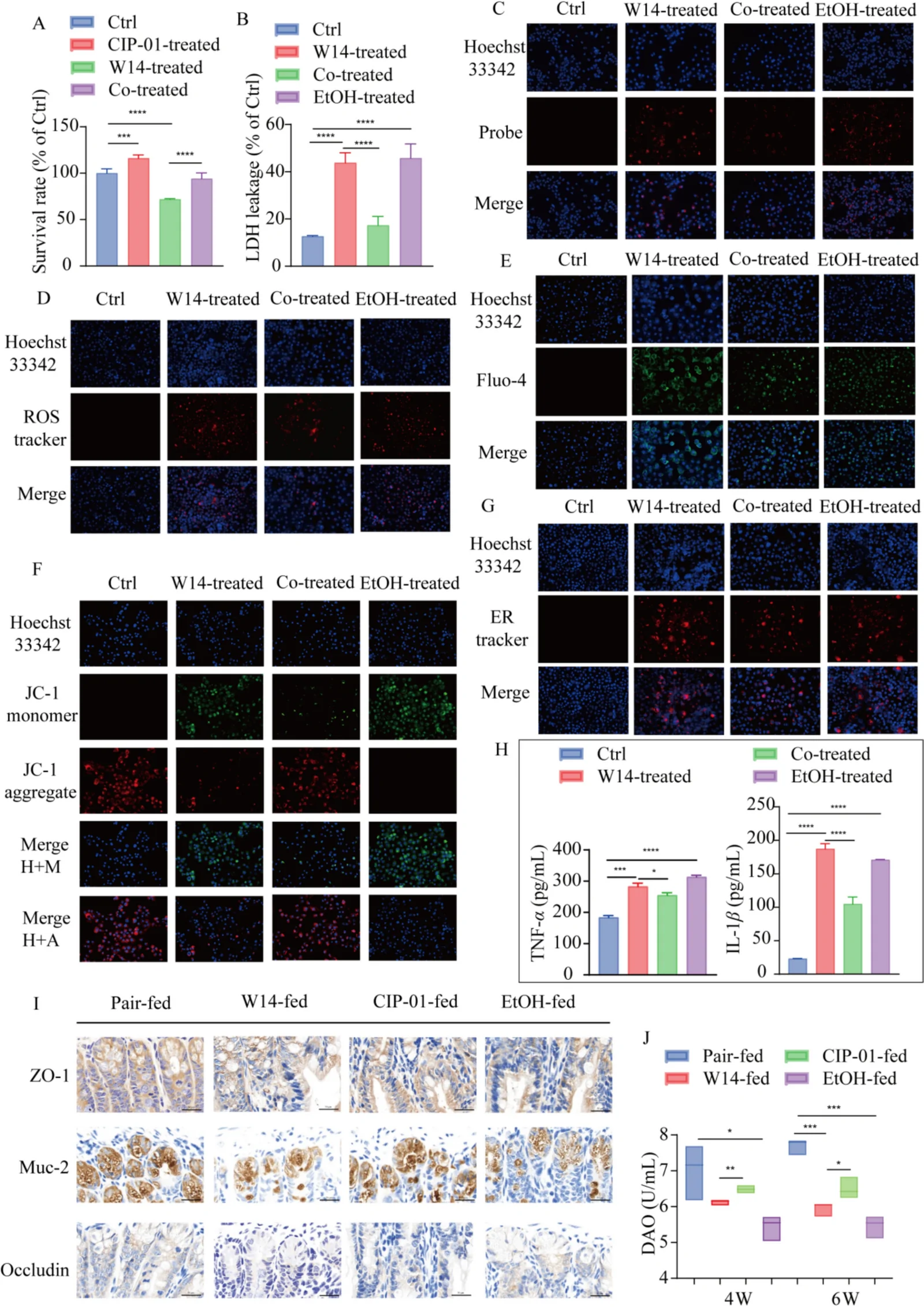
*B. bifidum* CIP-01 protects intestinal barrier from HiAlc *Kpn* W14-induced toxic damage in vitro and in vivo. **A** and **B** The effects of HiAlc *Kpn* W14 and *B. bifidum* CIP-01 on NCM460 cells are analyzed by CCK-8 assay (**A**) and the detection of lactate dehydrogenase concentration. **C**–**G** Immunofluorescence microscopy analysis (20X) in NCM460 cells on apoptosis by a Annexin V-PE probe (red) (**C**), on ROS by ROS tracker (red) (**D**), on calcium influx by a fluorescent probe Fluo-4 AM (green) (**E**), on mitochondrial morphology with Mito-tracker (green/red) (**F**), and on ER with ER-tracker (red) (**G**), and nucleus is counterstained with Hoechst33342 (blue), n = 5; scale bars are not shown for clarity. **H** The concentration of inflammatory cytokines TNF-*α* and IL-1*β* in the supernatant are detected by ELISA kits, n = 5. All the NCM460 cells are treated with HiAlc *Kpn* W14 (MOI = 10) alone, co-treated with W14 and supernatant of *B. bifidum* CIP-01 culture medium (50 *μL*), as well as EtOH (500 μM), respectively, for 24 h, n = 6. (I) ZO-1, MUC-2, and Occludin proteins in intestinal tissues of mice (n = 3) are evaluated by IHC (40X). (J) The serum level of DAO from SPF mice fed with HiAlc *Kpn* W14, *B. bifidum* CIP-01, EtOH and a chow diet for 4 and 6 weeks. All data are presented as the mean ± SD. *ns, P* > 0.05, ^*^P < *0.05*, ^**^P < *0.01*, ^***^P < 0.005, ^****^P < *0.001*. *P* value was assessed by two-way ANOVA

To elucidate the mechanism by which *B. bifidum* CIP-01 exerts cross-organ protection, we established an intestinal epithelial NCM460 cell injury model. The results showed that HiAlc *Kpn* inhibited NCM460 cell proliferation (Fig. 2A), increased lactate dehydrogenase release (Fig. 2B), and induced apoptosis (Fig. 2C). It also caused damage to colonic epithelial cells, characterized by elevated ROS levels (Fig. 2D), increased intracellular Ca^2+^ influx (Fig. 2E), enhanced endoplasmic reticulum stress (Fig. 2G), and reduced mitochondrial membrane potential (evidenced by stronger green fluorescence and weaker red fluorescence) (Fig. 2F). In addition, levels of the inflammatory cytokines TNF-*α* and IL-1*β* were elevated in culture supernatant (Fig. 2H).

Supplementation with *B. bifidum* CIP-01 supernatant substantially alleviated the cytotoxic effects induced by HiAlc *Kpn*. The supernatant promoted NCM460 cell proliferation; when HiAlc *Kpn* was co-incubated with *B. bifidum* CIP-01 supernatant, the inhibitory effect of *K. pneumoniae* on cell proliferation was considerably diminished (Fig. 2A). Other forms of cellular injury also were substantially reduced (Fig. 2D–G). These findings suggest that *B. bifidum* CIP-01 protects intestinal epithelial cells and preserves their function by reducing intracellular ROS accumulation through antioxidant mechanisms, including regulation of calcium homeostasis, attenuation of endoplasmic reticulum stress, and maintenance of mitochondrial function. These effects contribute to decreased intestinal inflammation (Fig. 2H).

Furthermore, intestinal permeability significantly increased. After infection with HiAlc *Kpn*, the expression level of mucin MUC-2, a key component responsible for intestinal barrier maintenance, was considerably reduced in mice (Fig. 2I). The expression levels of essential tight junction proteins, including ZO-1 and occludin, also were downregulated in intestinal tissues (Fig. 2I). These findings indicated substantial disruption of intestinal barrier integrity, further supported by a substantial decrease in serum DAO levels (Fig. 2J). In contrast, mice supplemented with *B. bifidum* CIP-01 exhibited partial restoration of intestinal permeability-related protein expression (Fig. 2J), along with improved serum DAO levels (Fig. 2I).

Taken together, these results suggest that metabolites derived from *B. bifidum* CIP-01 can modulate mitochondrial membrane potential and endoplasmic reticulum function, suppress intracellular ROS levels, and inhibit intestinal epithelial apoptosis. Such effects help preserve intestinal barrier integrity and prevent translocation of HiAlc *Kpn* and its metabolites to the liver, thereby protecting against hepatocyte injury.

### Hepatic protection by *B. bifidum* CIP-01 involves suppression of cytochrome P450 2E1 (CYP2E1)-mediated oxidative stress and lipid accumulation

**Fig. 3:**
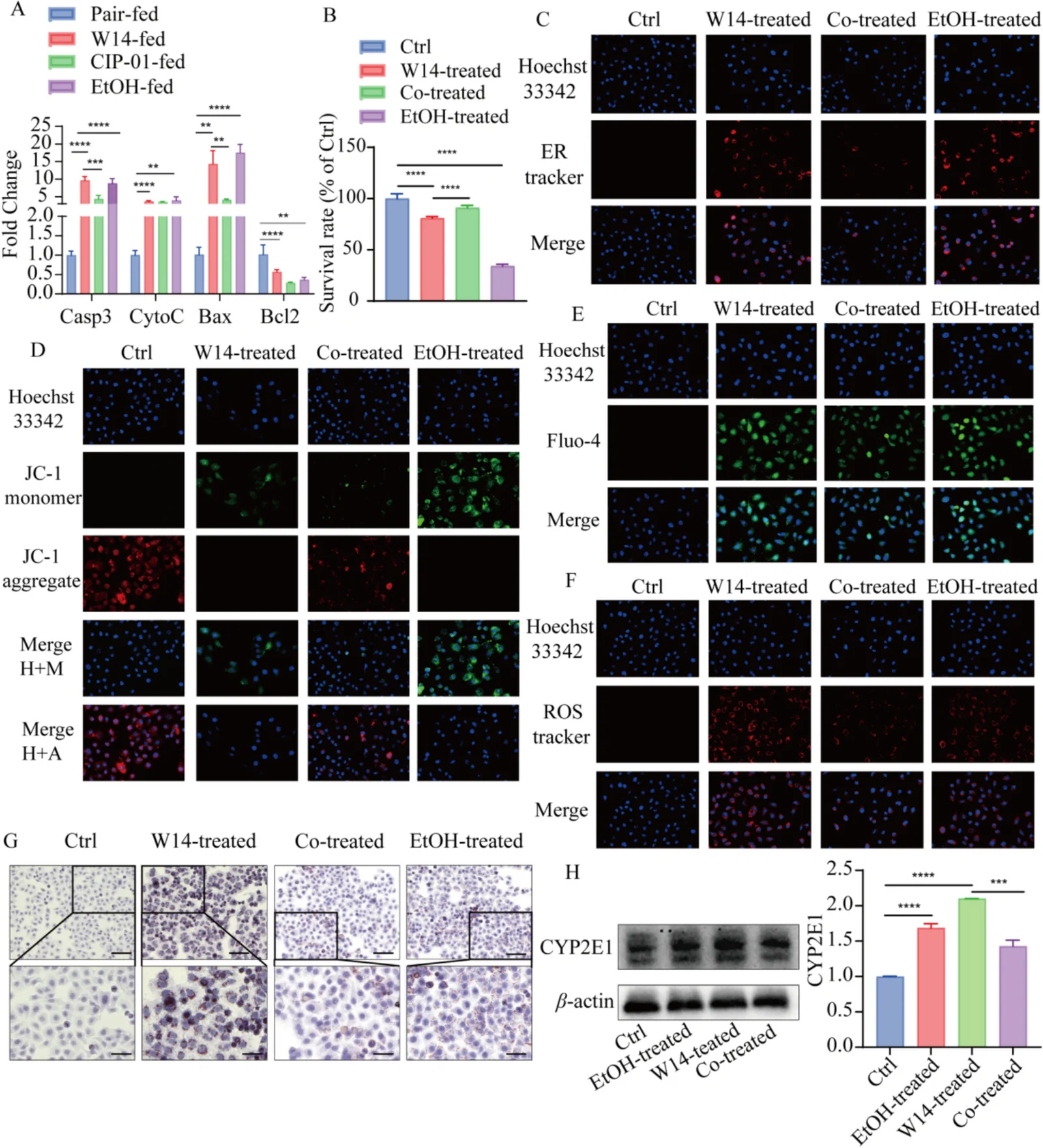
*B. bifidum* CIP-01 protects HepG2 cells against HiAlc *Kpn* W14 with metabolic activity. **A** Real-time PCR shows the expression of apoptosis-related genes in hepatocytes of mice, n = 3. **B** Effects of HiAlc *Kpn* W14 and *B. bifidum* CIP-01 on the proliferation of HepG2 cells with CCK-8 assay. **C**–**F** Immunofluorescence microscopy analysis (20X) in HepG2 cells on ER with ER tracker (red) (**C**), on mitochondrial morphology with Mito tracker (green/red) (**D**), on calcium influx by a fluorescent probe Fluo-4 AM (green) (E), and on ROS by ROS tracker (red) (**F**) are carried out, and nucleus is counterstained with Hoechst33342 (blue), n = 5; scale bars were not shown for clarity. **G** Representative ORO staining of HepG2 cells (20X). **H** Protein expression of CYP2E1 in HepG2 cells, n = 3. All the HepG2 cells are treated with HiAlc *Kpn* W14 (MOI = 10) alone, co-treated with W14 and supernatant of *B. bifidum* CIP-01 culture medium (50 *μL*), as well as EtOH (500 μM), respectively for 24 h, n = 6. All data are presented as the mean ± SD. *ns, P* > 0.05, ^*^P < *0.05*, ^**^P < *0.01*, ^***^P < 0.005, ^****^P < *0.001*. *P* value was assessed by two-way ANOVA

Quantitative analysis of hepatic gene expression in mice revealed that HiAlc *Kpn* W14 significantly upregulated apoptosis markers, including caspase-3, cytochrome c, and Bax. Supplementation with *B. bifidum* CIP-01 effectively inhibited activation of these apoptotic pathways (Fig. 3A). To further investigate the protective mechanism of *B. bifidum* CIP-01 in hepatocytes, we established an injury model using HepG2 cells. Compared with the control group, HiAlc *Kpn* suppressed cell proliferation (Fig. 3B), increased endoplasmic reticulum stress (Fig. 3C), decreased mitochondrial membrane potential (Fig. 3D), promoted Ca^2+^ influx (Fig. 3E), and elevated ROS levels (Fig. 3F). In addition, HiAlc *Kpn* W14 induced lipid accumulation in hepatocytes (Fig. 3G). These findings suggest that HiAlc *Kpn* W14 and/or its metabolites severely impair hepatocyte function, disrupt glucose and lipid metabolism, and promote intracellular lipid droplet deposition, consistent with in vivo observations (Fig. 1E). However, co-treatment with *B. bifidum* CIP-01 supernatant substantially mitigated hepatocyte cytotoxicity. This protective effect was characterized by restoration of mitochondrial membrane potential, maintenance of endoplasmic reticulum synthetic function, suppression of ROS release, and inhibition of apoptotic signaling pathways, collectively supporting the regulation of intracellular lipid metabolism (Fig. 3A–F).

CYP2E1 is a key metabolic enzyme in the liver, primarily involved in the oxidative metabolism of exogenous substances such as ethanol and various drugs. A high expression level of CYP2E1 was observed in HepG2 cells during HiAlc *Kpn* infection and under ethanol stimulation. However, the CYP2E1 protein level rapidly decreased upon treatment with *B. bifidum* CIP-01 supernatant (Fig. 3H).

Our results indicate that elevated CYP2E1 expression may result from a localized high concentration of ethanol and other toxins generated by HiAlc *Kpn* during its metabolic activity. This condition likely exacerbates ROS production and oxidative damage to hepatocytes, ultimately leading to necrosis or apoptosis. In contrast, *B. bifidum* CIP-01 supernatant appears to attenuate oxidative hepatocellular injury by modulating CYP2E1 expression.

### *B. bifidum* CIP-01 modulates gut microbiota disrupted by HiAlc *Kpn* W14

To evaluate the impact of *B. bifidum* CIP-01 on gut microbiota composition, we performed 16S rRNA sequencing on fecal samples from experimental mice. Analysis of microbial diversity revealed that administration of HiAlc *Kpn* significantly reduced the *α*-diversity index of the gut microbiota compared with the control group, as indicated by a decrease in the Shannon index (*P* < 0.01) (Fig. S5A). This reduction suggests disruption of the microbial ecosystem, potentially due to competitive exclusion or metabolic interference, and the formation of a synergistic pathogenic pattern with ethanol metabolites produced by W14 strains, which directly impair intestinal barrier function. Analysis of the relative abundances of bacterial genera identified significant differences among treatment groups. Compared with the pair-fed group, HiAlc *Kpn* administration altered the abundances of several genera. Notably, the relative abundances of *Streptococcus*, *Enterococcus*, and *Allobaculum* increased in the HiAlc *Kpn*-fed group; the abundance of *Coprococcus*, an SCFA-producing genus with known anti-inflammatory properties, was decreased.

**Fig. 4:**
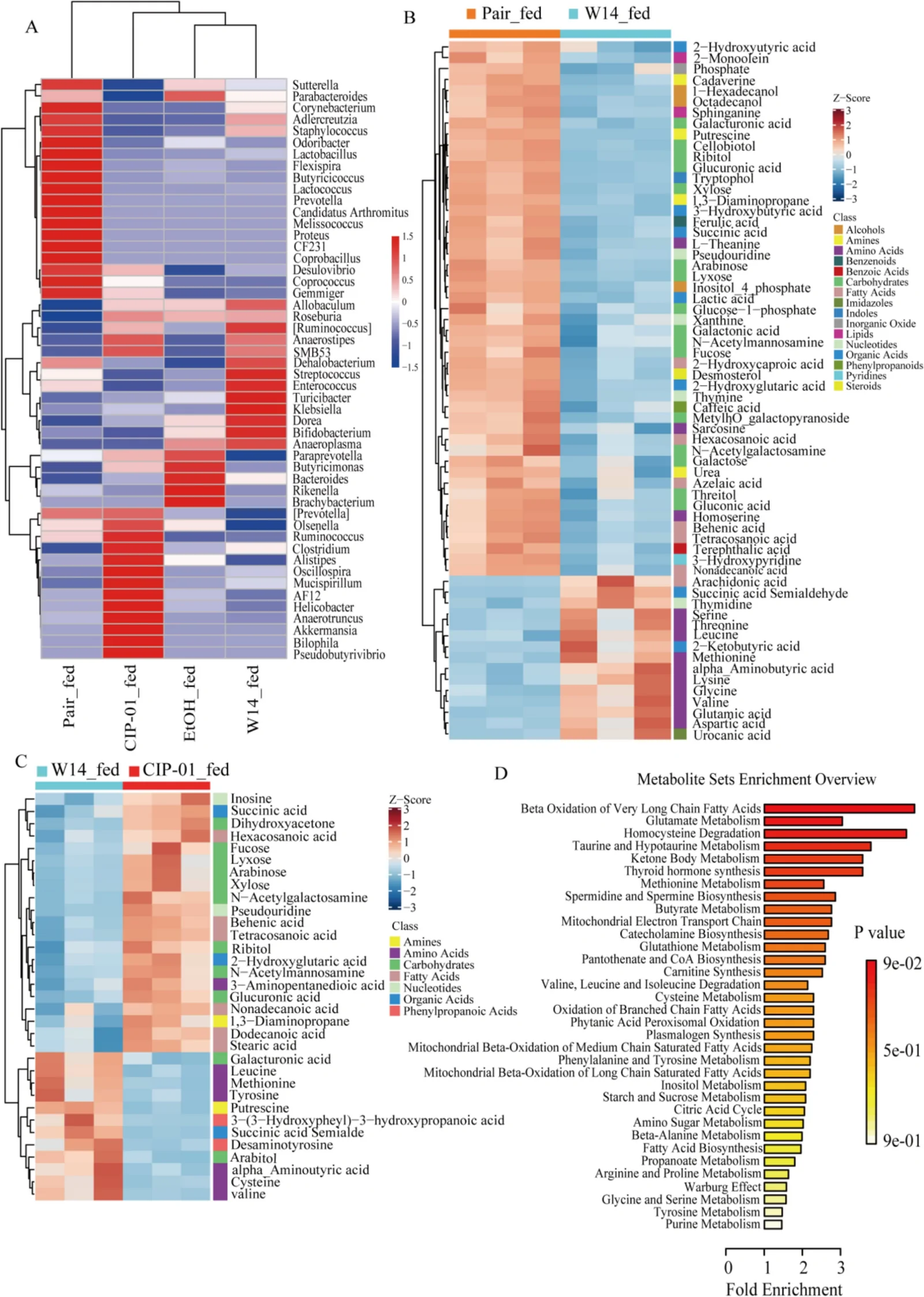
*B. bifidum* CIP-01 alters the metabolic profile of MASLD mice induced by HiAlc *Kpn* W14. **A** Heatmap showing the compositions of intestinal microbiota in feces of experimental mice fed with HiAlc *Kpn* W14, *B. bifidum* CIP-01, EtOH and a chow diet for 4 and 6 weeks. **B** and **C** Heatmaps showing the differential metabolites between pair-fed *vs.* HiAlc *Kpn* W14-fed mice (**B**), and HiAlc *Kpn* W14-fed mice *vs. B. bifidum* CIP-01-fed mice (**C**). **D** The metabolic pathways in HiAlc *Kpn* W14-fed mice *vs. B. bifidum* CIP-01-fed mice

In mice supplemented with *B. bifidum* CIP-01, the species diversity in the fecal samples has been significantly restored (Fig. S5A). What's more, the abundances of pathogenic genera such as *Streptococcus*, *Enterococcus*, and *Allobaculum* significantly decreased, whereas the abundances of *Akkermansia* and *Coprococcus* increased. Notably, the abundance of *K. pneumoniae* in fecal samples also significantly declined (Fig. 4A). Intriguingly, the abundance of the genus *Bifidobacterium* did not increase (Fig. 4A), suggesting that *B. bifidum* CIP-01 was unable to stably colonize the mouse intestine under specific pathogen-free conditions.

These results indicate that HiAlc *Kpn* W14 disrupted gut microbial homeostasis. Although *B. bifidum* CIP-01 did not enhance the abundances of probiotic genera such as *Lactobacillus* and *Lactococcus*, its administration reduced the abundance of pathogenic bacteria induced by HiAlc *Kpn*, partially restored microbial balance, and improved gut microbiota homeostasis. This effect appeared to result from metabolic regulation-driven microbiota remodeling, rather than stable colonization by *B. bifidum* CIP-01.

### *B. bifidum* CIP-01 reverses toxicity induced by HiAlc *Kpn* W14 via metabolic regulation

To further investigate the mechanisms by which *B. bifidum* CIP-01 protects the intestinal barrier and alleviates liver injury, we performed metabolomic analyses on fecal samples from experimental mice. Compared with the pair-fed group, HiAlc *Kpn* W14 induced metabolic disturbances, primarily characterized by pro-inflammatory lipid accumulation, amino acid imbalance, and impaired antioxidant defense. Substantial alterations in key metabolites were observed. Arachidonic acid (a pro-inflammatory precursor), succinic acid (which activates hypoxia inducible factor-1*α*), branched-chain amino acids (BCAAs; e.g., leucine, valine), and uric acid (which activates NOD-like receptor family pyrin domain containing 3) were upregulated. Conversely, choline (a protective factor for the intestinal barrier), malic acid (a tricarboxylic acid cycle intermediate), galacturonic acid (a component of the intestinal mucus layer), and organic acids (e.g., lactic acid, gluconic acid) were downregulated (Fig. 4B). Supplementation with *B. bifidum* CIP-01 significantly reversed the metabolic toxicity induced by W14. Anti-inflammatory and antioxidant metabolites were upregulated, whereas pro-inflammatory and injury-related metabolites were downregulated (Fig. 4C). Specifically, succinic acid, galacturonic acid, and amino acids such as leucine, valine, and methionine were modulated. In addition, several protective metabolites appeared, including inosine (with anti-inflammatory properties), mannose (which inhibits pathogen adhesion), N-acetylgalactosamine (beneficial for mucus repair), and glucuronic acid (involved in detoxification).

Enrichment analysis of differential metabolites showed that HiAlc *Kpn* significantly enriched pathways related to BCAA catabolism, energy metabolism, and the clearance of toxic intermediates, including enzymes such as L-threonine deaminase and methylmalonate semialdehyde dehydrogenase (Fig. S5B). Compared with the group exposed to HiAlc *Kpn* W14 alone, the group supplemented with *B. bifidum* CIP-01 exhibited reduced BCAA degradation pathways and significant enrichment in propionate and butyrate metabolism (Fig. 4D). Additional enriched pathways included Warburg effect-related mechanisms, mitochondrial long-chain and medium-chain fatty acid *β*-oxidation, fatty acid biosynthesis, glutathione metabolism, and plasmalogen synthesis. Metabolomic analysis of in vitro-cultured *B. bifidum* CIP-01 revealed significantly increased levels of SCFAs, including propionic acid (hydroxypropionic acid), butyric acid (3-hydroxybutyric acid, 2-hydroxybutyric acid), and caproic acid, as well as other organic acids (e.g., lactic, succinic, fumaric, and malic acids) (Fig. S6A–B). These findings suggest that the protective effect of *B. bifidum* CIP-01 involves regulation of the host metabolic microenvironment.

### *B. bifidum* CIP-01 supernatant disrupts the proliferation–biofilm axis to attenuate virulence of HiAlc *Kpn* W14

**Fig. 5:**
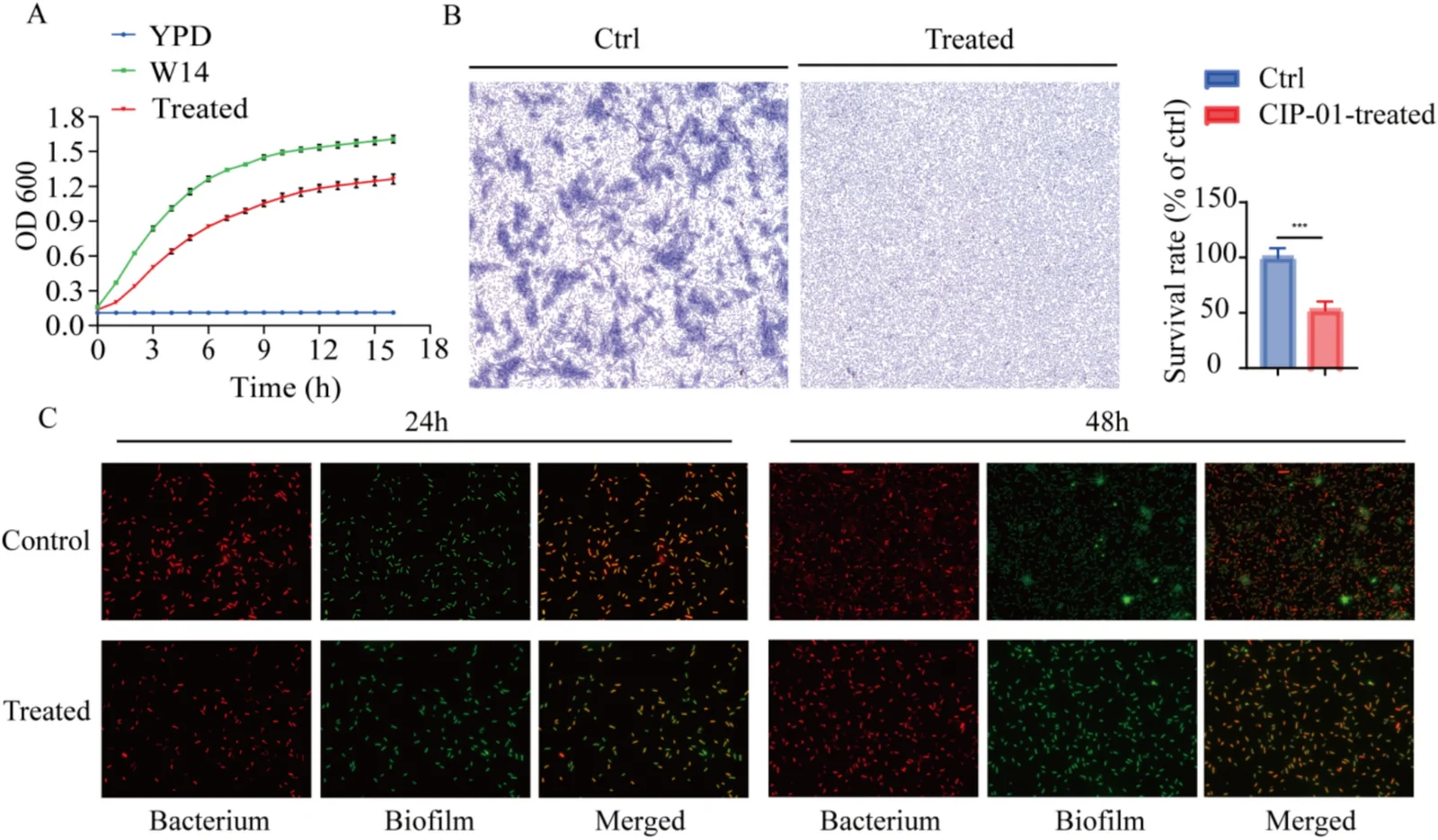
*B. bifidum* CIP-01 shows a negative effect on HiAlc *Kpn* W14. **A** and **B** Effect of *B. bifidum* CIP-01 on growth of HiAlc *Kpn* W14 (**A**), microscopic analysis (20X) with crystal violet staining and absorbance quantification (B), n = 6. **C** Images of biofilm formation (40X). HiAlc *Kpn* W14 are treated with supernatant of *B. bifidum* CIP-01 culture medium (treated at 20% v/v), n = 6. All data are presented as the mean ± SD. ^***^P < *0.005 vs.* control. *P* value was assessed by two-tailed Student’s *t* test

The supernatant of *B. bifidum* CIP-01 exhibited a strong inhibitory effect on the pathogenicity of HiAlc *Kpn* W14. In vitro experiments demonstrated that treatment with *B. bifidum* CIP-01 supernatant (20% v/v for 24 h) significantly suppressed W14 proliferation, with a maximum inhibition rate of 47.7% (*P* < 0.005) (Fig. 5A-B). Concurrently, the biofilm-forming ability of the W14 strain was substantially reduced (Fig. 5C). What's more, the transwell co-culture assay indicated that even in the absence of niche competition, CIP-01 strain can directly inhibit the proliferation of W14 through its metabolites (Fig. S7B). These results indicate that *B. bifidum* CIP-01 metabolites effectively neutralizes the toxic effects of HiAlc *Kpn* by simultaneously targeting two critical pathogenic axes: bacterial proliferation and biofilm formation. This dual mechanism suggests that metabolites of *B. bifidum* CIP-01 have utility in anti-virulence therapeutic applications.

## Discussion

**Fig. 6:**
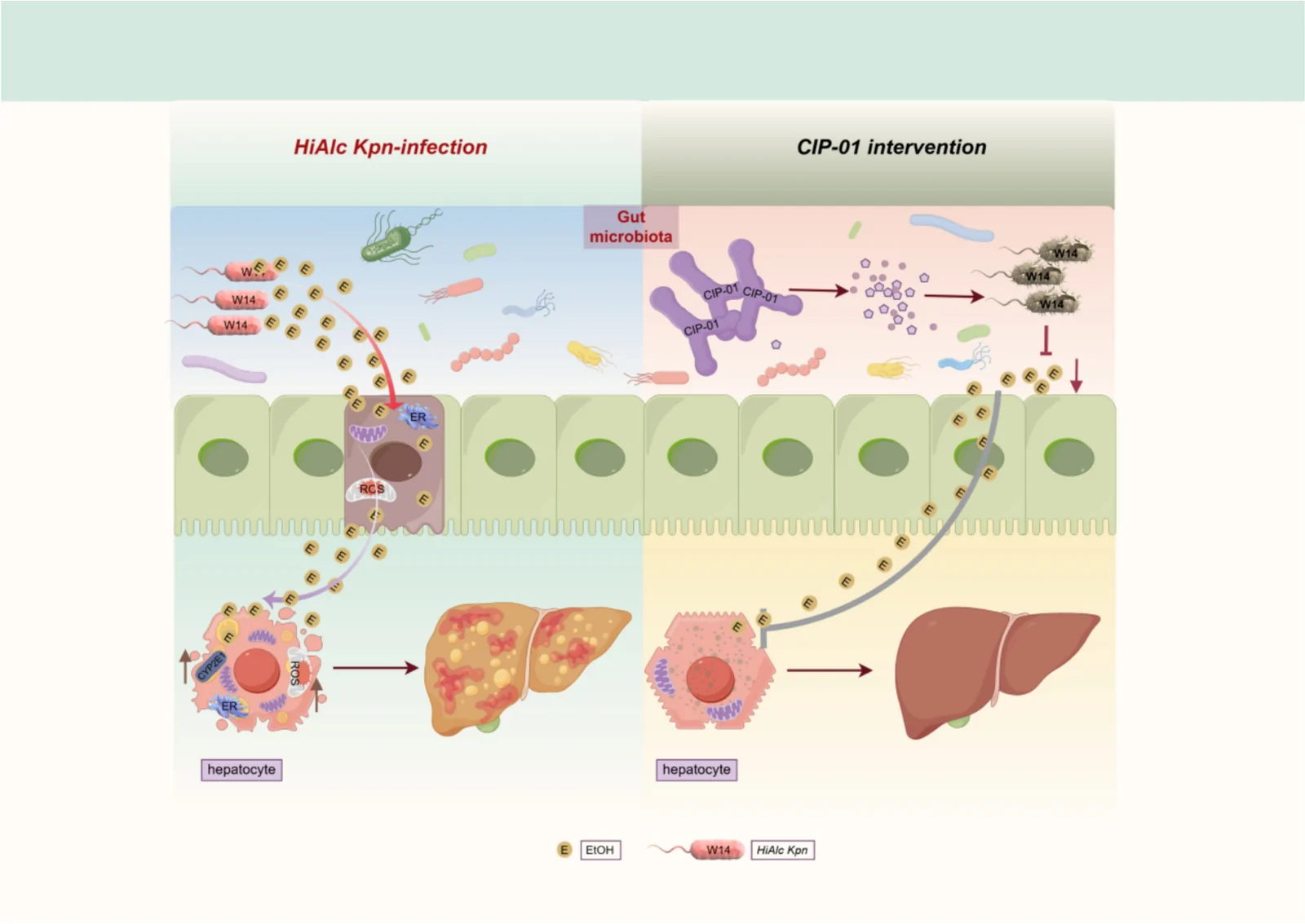
Graphical abstract by figdraw.com

This study demonstrated that *B. bifidum* CIP-01 inhibits the virulence of HiAlc *Kpn* W14 through a dual mechanism of direct antagonism and metabolic intervention (Fig. 6), providing key mechanistic support for its protective effects against intestinal and hepatic injury. During in vitro experiments, *B. bifidum* CIP-01 supernatant significantly inhibited the proliferation of HiAlc *Kpn* W14 and reduced its biofilm-forming capacity, suggesting that *B. bifidum* CIP-01 directly targets bacterial virulence factors through secretion of antibacterial metabolites. This anti-virulence strategy—rather than direct bactericidal action—resembles the mechanism by which other lactic acid bacteria (e.g., *Lactobacillus* species) inhibit intestinal pathogens via bacteriocin production [21]. Previous studies have shown that *B. bifidum* CIP-01 produces metabolites such as acetate and lactic acid, which suppress the growth of Gram-negative bacteria, including *Escherichia coli* [22, 23]. Metabolomic analysis of *B. bifidum* CIP-01 revealed significantly elevated levels of SCFAs, including hydroxypropionic, 3-hydroxybutyric, 2-hydroxybutyric, and caproic acids (Fig. S6A–B), suggesting a mechanism of action that involves acidification of the intestinal environment and consequent inhibition of HiAlc *Kpn* W14 colonization. This inference is supported by microbiota sequencing, which showed a substantial reduction in *K. pneumoniae* abundance after *B. bifidum* CIP-01 administration (Fig. 4B). This finding is consistent with previous reports indicating that butyric acid can inhibit *Klebsiella* pathogenicity by downregulating virulence genes, such as the biofilm-associated gene *mrkA* [24]. Furthermore, high concentrations of organic acids—including lactic, succinic, fumaric, and malic acids—were enriched in the in vitro metabolomic profile of *B. bifidum* CIP-01 (Fig. S6A). These metabolites participate in glycolysis and the tricarboxylic acid cycle; their accumulation may competitively consume carbon sources required by pathogenic bacteria. In addition, as a weak organic acid, lactic acid can penetrate bacterial cell membranes and dissociate into H^+^ and lactate ions, leading to intracellular acidosis in W14 strains. This process inhibits adenosine triphosphate production and suppresses bacterial proliferation. Furthermore, the ecological competition effect regulated by *B. bifidum* CIP-01 may indirectly restrict the expansion of HiAlc *Kpn* by optimizing the microbial community structure. For example, after *B. bifidum* CIP-01 supplementation, the abundances of genera such as *Akkermansia* were restored (Fig. 4A), potentially weakening the ecological advantage of HiAlc *Kpn* W14 through competition for adhesion sites or carbon sources. This reconfiguration of the probiotic–pathogen interaction network complements the mechanism by which propionic acid induces host antimicrobial peptide expression (e.g., Reg3*γ*), enhancing antibacterial defenses [25]. These processes jointly establish a multidimensional prevention and control system against HiAlc *Kpn* W14. Notably, this study demonstrated that *B. bifidum* CIP-01 blocks the expansion of intestinal pathogens through a combination of metabolic targeted intervention (inhibiting pathogenic proliferation) and niche replacement (promoting beneficial colonization), thus offering a new perspective for the development of microecological therapies that target alcohol-producing enteric bacteria associated with liver disease.

Moreover, this study systematically elucidated the core mechanisms by which *B. bifidum* CIP-01 restores gut–liver axis homeostasis through multidimensional regulation of microbial metabolites and host barrier functions. Intestinal permeability, as evidenced by decreased serum DAO levels, increased after HiAlc *Kpn* W14 colonization. The resulting transfer of HiAlc *Kpn* metabolites (primarily ethanol) to the liver directly triggers CYP2E1-dependent oxidative damage (Fig. 3H), a pathological process consistent with the known mechanism by which intestinal-derived endotoxins trigger hepatic inflammation via the portal vein in the MASLD model [26, 27]. Intervention with *B. bifidum* CIP-01 effectively interrupts this vicious cycle through a multifaceted repair strategy: a) reinforcement of the mucus barrier: *B. bifidum* CIP-01 significantly increases MUC-2 expression in the colon (Fig. 2I). This effect may be related to the enrichment of N-acetylgalactosamine—a precursor for glycoprotein synthesis—in the metabolome (Fig. 4C), thereby enhancing the physical barrier function of the mucus layer against pathogens [28, 29]. b) Regulation of energy metabolism: SCFAs (hydroxypropionic, 3-hydroxybutyric, 2-hydroxybutyric, and caproic acids) inhibit myosin light-chain phosphorylation through activation of the adenosine monophosphate-activated protein kinase/myosin light-chain kinase pathway. This inhibition modulates cellular and energy metabolism [30–32], while restoring expression of the tight junction proteins ZO-1 and occludin (Fig. 2I). c) Immune regulation mediated by microbial metabolites: *B. bifidum* CIP-01 increases glutathione levels (Fig. 4D), reduces mitochondrial ROS in intestinal epithelial cells (Fig. 2D), and substantially attenuates endoplasmic reticulum stress (Fig. 2G). This intervention also restores mitochondria–endoplasmic reticulum interaction homeostasis (Fig. 2F), as indicated by recovery of JC-1 fluorescence, and inhibits endoplasmic reticulum stress-induced intestinal epithelial apoptosis (Fig. 2C). These findings suggest that *B. bifidum* CIP-01 extends beyond the single-barrier repair mechanism typically attributed to probiotics. Through regulating of the metabolome—including SCFAs, amino acids, and antioxidant molecules—it coordinately regulates energy metabolism (e.g., the Warburg effect, Fig. 4C) and maintains organelle homeostasis in epithelial cells. In addition, microbiota sequencing results (Fig. S6A) indicate that increased levels of propionic and butyric acid may suppress the NF-*κ*B pathway by activating free fatty acid receptors 2 and 3 on intestinal endocrine cells [33–35]. This action reduces TNF-*α* and interleukin-1*β* expression in intestinal epithelial cells (Fig. 2H), forming a bidirectional regulatory network between microbial metabolites and host immunity. Collectively, these mechanisms explain the unique advantage of *B. bifidum* CIP-01 in simultaneously restoring intestinal barrier integrity and attenuating hepatic injury in the MASLD model.

Finally, metabolic remodeling represents a central mechanism by which *B. bifidum* CIP-01 reverses liver injury. HiAlc *Kpn* W14 activates the catabolism of BCAAs, such as valine and isoleucine, substantially enriching pathways involving mitochondrial L-3-amino-isobutyric acid exchange and 2-hydroxybutyrate: NAD^+^ oxidoreductase. This activation leads to ROS accumulation, lipid deposition in hepatocytes, and upregulation of CYP2E1 expression. *B. bifidum* CIP-01 mitigates this damage through metabolic intervention: a) restoration of mitochondrial fatty acid oxidation: *B. bifidum* CIP-01 supplementation significantly enriches pathways related to mitochondrial long-chain and medium-chain saturated fatty acid *β*-oxidation and carnitine synthesis, the latter facilitating fatty acid transport into mitochondria. These changes, along with activation of the tricarboxylic acid cycle and mitochondrial electron transport chain (Fig. 4D), provide a molecular basis for the observed improvement in mitochondrial membrane potential (as indicated by increased JC-1 aggregate formation, Fig. 3D) and reversal of hepatic lipid accumulation (Fig. 3G) after treatment with *B. bifidum* CIP-01 supernatant. b) Membrane-protective effect of plasmalogen: The *B. bifidum* CIP-01-treated group showed robust activation of the plasmalogen synthesis pathway, including upregulation of key ether lipid synthesis enzymes. This enhancement strengthens mitochondrial membrane resistance to lipid peroxidation, consistent with the observed reduction in lipid ROS among HepG2 cells treated with *B. bifidum* CIP-01 supernatant (Fig. 3F). The therapeutic efficacy of *B. bifidum* CIP-01 depends on comprehensive restructuring of gut microbiota composition and function. Its intervention strongly suppresses opportunistic pathogens such as *Klebsiella*, promotes the proliferation of beneficial genera including *Akkermansia*, and increases the abundance of anti-inflammatory metabolites (e.g., butyric acid and inosine) (Fig. 4A). This microbial profile sharply contrasts with that observed among patients with alcoholic hepatitis [36], where *Enterobacteriaceae* dominate and pro-inflammatory metabolites such as succinic acid and arachidonic acid accumulate. Importantly, *B. bifidum* CIP-01 may establish a synergistic microbial network through metabolic cross-feeding, such as acetate production supporting the growth of butyrate-producing bacteria. This ecological feature offers new insights for the targeted modulation of gut microbiota.

## Conclusion

Overall, this study elucidates the pathological mechanism by which *K. pneumoniae* W14, a high-alcohol-producing strain, disrupts microbiota structure and metabolic balance, leading to oxidative stress and hepatic lipid accumulation. It also defines the intervention pathway through which *B. bifidum* CIP-01 restores energy homeostasis by reprogramming the host metabolic network. These findings underscore the unique advantages of *B. bifidum* CIP-01 as a metabolic homeostasis regulator. Through bidirectional modulation of BCAA catabolism and mitochondrial lipid oxidation, it simultaneously alleviates oxidative stress and lipid metabolic dysfunction in both intestinal and hepatic tissues. The mechanism of *B. bifidum* CIP-01 involves multilevel reconstruction of the host metabolic landscape through a coordinated axis of metabolic regulation, energy optimization, and gut barrier protection. This distinguishes it from established probiotic mechanisms that predominantly focus on SCFA production, offering a novel theoretical framework for microbiota-targeted interventions in metabolic syndrome. Moreover, the functional activity of *B. bifidum* CIP-01 is mediated by secreted metabolites, rather than through colonization by viable bacteria. This observation, consistent with previous studies [20], supports the development of microecological formulations such as post-biotics derived from *B. bifidum* CIP-01 metabolites. These formulations may be especially suitable for immunocompromised patients who must avoid potential risks associated with live bacterial interventions.

### Limitations of the study

While this study provides valuable insights into the therapeutic potential of *B. bifidum* CIP-01 in HiAlc *Kpn*-induced MASLD, several limitations should be acknowledged. First, although we employed a well-established W14 strain colonization model, we did not measure portal vein ethanol levels, which could have provided direct evidence linking gut-derived ethanol to hepatic pathology. Second, the current 4–8 week intervention period precluded assessment of fibrotic progression and long-term hepatoprotective effects (> 12 weeks) of CIP-01. Third, our exclusive focus on HiAlc *Kpn*-driven MASLD limits extrapolation to other MASLD subtypes; future studies should evaluate CIP-01’s efficacy in broader metabolic contexts. In addition, while our IHC quantification methods (averaging multiple fields per animal) were rigorous, the modest group size (n = 3) may constrain detection of subtle effects. Larger validation cohorts will be essential to confirm these findings and further elucidate the translational potential of microbiota-targeted therapies across heterogeneous MASLD populations.

## Supplementary Information

Below is the link to the electronic supplementary material.

Supplementary file1 (DOCX 26524 KB)

## References

[1] Sakurai Y, Kubota N, Yamauchi T, et al. Role of insulin resistance in MAFLD. Int J Mol Sci. 2021. 10.3390/ijms22084156. PMC8072900 33923817

[2] Rutledge SM, Soper ER, Ma N, et al. Association of HSD17B13 and PNPLA3 with liver enzymes and fibrosis in hispanic/latino individuals of diverse genetic ancestries. Clin Gastroenterol Hepatol. 2023;10:2578–87. 10.1016/j.cgh.2022.12.025. 36610497

[3] Han CY, Rho HS, Kim A, et al. FXR inhibits endoplasmic reticulum stress-induced NLRP3 inflammasome in hepatocytes and ameliorates liver injury. Cell Rep. 2018. 10.1016/j.celrep.2018.07.068. 30208322

[4] Fernandez-Cantos MV, Garcia-Morena D, Iannone V, et al. Role of microbiota and related metabolites in gastrointestinal tract barrier function in NAFLD. Tissue Barriers. 2021. 10.1080/21688370.2021.1879719. PMC8489918 34280073

[5] Chopyk DM, Grakoui A. Contribution of the intestinal microbiome and gut barrier to hepatic disorders. Gastroenterology. 2020. 10.1053/j.gastro.2020.04.077. PMC7502510 32569766

[6] Iacob S, Iacob DG. Infectious threats, the intestinal barrier, and its Trojan horse: dysbiosis. Front Microbiol. 2019;10:1676. 10.3389/fmicb.2019.01676. PMC6692454 31447793

[7] Wang X, Fang Y, Liang W, et al. Gut-liver translocation of pathogen *Klebsiella pneumoniae* promotes hepatocellular carcinoma in mice. Nat Microbiol. 2025. 10.1038/s41564-024-01890-9. PMC11726454 39747695

[8] Nakamoto N, Sasaki N, Aoki R, et al. Gut pathobionts underlie intestinal barrier dysfunction and liver T helper 17 cell immune response in primary sclerosing cholangitis. Nat Microbiol. 2019. 10.1038/s41564-018-0333-1. 30643240

[9] Yuan J, Chen C, Cui J, et al. Fatty liver disease caused by high-alcohol-producing *Klebsiella pneumoniae*. Cell Metab. 2019;4:675-88.e7. 10.1016/j.cmet.2019.08.018. 31543403

[10] Gan L, Feng Y, Du B, et al. Bacteriophage targeting microbiota alleviates non-alcoholic fatty liver disease induced by high alcohol-producing *Klebsiella pneumoniae*. Nat Commun. 2023. 10.1038/s41467-023-39028-w. PMC10239455 37270557

[11] Zhang R, Xu Z, Xue G, et al. Combined methylation and transcriptome analysis of liver injury of nonalcoholic fatty liver disease induced by high alcohol-producing *Klebsiella pneumoniae*. Microbiol Spectr. 2023;11(3):e0532322. 10.1128/spectrum.05323-22 PMC10269619 37022192

[12] Shen M, Zhao H, Han M, et al. Alcohol-induced gut microbiome dysbiosis enhances the colonization of *Klebsiella pneumoniae *on the mouse intestinal tract. mSystems. 2024. 10.1128/msystems.00052-24. PMC10949497 38345382

[13] Wang J, Zhang Z, Wang J, et al. *Bacillus coagulans* alleviates intestinal barrier injury induced by *Klebsiella pneumoniae* in rabbits by regulating the TLR4/MyD88/NF-*κ*B signalling pathway. Vet Microbiol. 2025;301:110364. 10.1016/j.vetmic.2024.110364. 39755051

[14] Gomes AC, et al. Gut microbiota, probiotics and diabetes. Nutr J. 2014;13:60. 10.1186/1475-2891-13-60. PMC4078018 24939063

[15] Xiong M, Sun W. Research progress of probiotics and their protective strategy in the field of inflammatory bowel disease treatment: a review. Medicine (Baltimore). 2024. 10.1097/MD.0000000000040401. PMC11537665 39495980

[16] Wang L, Jiao T, Yu Q, et al. *Bifidobacterium bifidum* shows more diversified ways of relieving non-alcoholic fatty liver compared with *Bifidobacterium adolescentis*. Biomedicines. 2021. 10.3390/biomedicines10010084. PMC8772902 35052765

[17] Iliopoulos D, Drosatos K, Hiyama Y, et al. MicroRNA-370 controls the expression of microRNA-122 and Cpt1alpha and affects lipid metabolism. J Lipid Res. 2010;51:1513–23. 10.1194/jlr.M004812. PMC3035515 20124555

[18] Preising J, Philippe D, Gleinser M, et al. Selection of bifidobacteria based on adhesion and anti-inflammatory capacity in vitro for amelioration of murine colitis. Appl Environ Microbiol. 2010;76:3048–51. 10.1128/AEM.03127-09. PMC2863435 20228095

[19] Philippe D, Heupel E, Blum-Sperisen S, et al. Treatment with *Bifidobacterium bifidum* 17 partially protects mice from Th1-driven inflammation in a chemically induced model of colitis. Int J Food Microbiol. 2011;149:45–9. 10.1016/j.ijfoodmicro.2010.12.020. 21257218

[20] Grimm V, Radulovic K, Riedel CU. Colonization of C57BL/6 mice by a potential probiotic *Bifidobacterium bifidum* strain under germ-free and specific pathogen-free conditions and during experimental colitis. PLoS ONE. 2015;10:e0139935. 10.1371/journal.pone.0139935. PMC4595203 26439388

[21] Anjana TSK. Bacteriocin-producing probiotic lactic acid bacteria in controlling dysbiosis of the gut microbiota. Front Cell Infect Microbiol. 2022;12:851140. 10.3389/fcimb.2022.851140 PMC9149203 35651753

[22] Ibrahim SA, Bezkorovainy A. Inhibition of *Escherichia coli* by *Bifidobacteria*. J Food Prot. 1993;56:713–5. 10.4315/0362-028X-56.8.713. 31113093

[23] Mogna L, Del Piano M, Deidda F, et al. Assessment of the in vitro inhibitory activity of specific probiotic bacteria against different *Escherichia coli* strains. J Clin Gastroenterol. 2012;46:S29-32. 10.1097/MCG.0b013e31826852b7. 22955353

[24] ElBaradei A, Yakout MA. *Stenotrophomonas maltophilia*: genotypic characterization of virulence genes and the effect of ascorbic acid on biofilm formation. Curr Microbiol. 2022;79:180. 10.1007/s00284-022-02869-7. PMC9068641 35508743

[25] Choi SM, McAleer JP, Zheng M, et al. Innate Stat3-mediated induction of the antimicrobial protein Reg3*γ* is required for host defense against MRSA pneumonia. J Exp Med. 2013. 10.1084/jem.20120260. PMC3600913 23401489

[26] Bibbò S, Ianiro G, Dore MP, et al. Gut microbiota as a driver of inflammation in nonalcoholic fatty liver disease. Mediators Inflamm. 2018;2018:9321643. 10.1155/2018/9321643. PMC5833468 29563854

[27] Fianchi F, Liguori A, Gasbarrini A, et al. Nonalcoholic fatty liver disease (NAFLD) as model of gut-liver axis interaction: from pathophysiology to potential target of treatment for personalized therapy. Int J Mol Sci. 2021;22:6485. 10.3390/ijms22126485. PMC8233936 34204274

[28] Fiete D, Beranek M, Baenziger JU. Molecular basis for protein-specific transfer of N-acetylgalactosamine to N-linked glycans by the glycosyltransferases *β*1,4-N-acetylgalactosaminyl transferase 3 (*β*4GalNAc-T3) and *β*4GalNAc-T4. J Biol Chem. 2012. 10.1074/jbc.M112.371567. PMC3436590 22722937

[29] Combret V, Rincé I, Budin-Verneuil A, et al. Utilization of glycoprotein-derived N-acetylglucosamine-L-asparagine during *Enterococcus faecalis* infection depends on catabolic and transport enzymes of the glycosylasparaginase locus. Res Microbiol. 2024. 10.1016/j.resmic.2023.104169. 37977353

[30] Iannucci LF, Sun J, Singh BK, et al. Short chain fatty acids induce UCP2-mediated autophagy in hepatic cells. Biochem Biophys Res Commun. 2016. 10.1016/j.bbrc.2016.10.072. 27773823

[31] Frampton J, Murphy KG, Frost G, et al. Short-chain fatty acids as potential regulators of skeletal muscle metabolism and function. Nat Metab. 2020. 10.1038/s42255-020-0188-7. 32694821

[32] Yu H, Li R, Huang H, et al. Short-chain fatty acids enhance the lipid accumulation of 3T3-L1 cells by modulating the expression of enzymes of fatty acid metabolism. Lipids. 2018. 10.1002/lipd.12005. 29488641

[33] Higashimura Y, Naito Y, Takagi T, et al. Propionate promotes fatty acid oxidation through the up-regulation of peroxisome proliferator — Activated receptor *α* in intestinal epithelial cells. J Nutr Sci Vitaminol (Tokyo). 2015;6:511–5. 10.3177/jnsv.61.511. 26875495

[34] Yang Y, Huang J, Li J, et al. The effects of butyric acid on the differentiation, proliferation, apoptosis, and autophagy of IPEC-J2 cells. Curr Mol Med. 2020;4:307–17. 10.2174/1566524019666191024110443. (**PMID: 31749427**). 31749427

[35] Huang J, Sun Z, Zhang G, et al. Ferulic acid mediates microbial fermentation of arabinoxylan to enhance host immunity by suppressing TLR4/NF-κB signaling. Int J Biol Macromol. 2025;298:139810. 10.1016/j.ijbiomac.2025.139810. 39814295

[36] Ciocan D, Rebours V, Voican CS, et al. Characterization of intestinal microbiota in alcoholic patients with and without alcoholic hepatitis or chronic alcoholic pancreatitis. Sci Rep. 2018;1:4822. 10.1038/s41598-018-23146-3. PMC5859299 29555983

