# Supplementary material for: Bifidobacterium bifidum CIP-01 attenuates metabolic dysfunction-associated steatotic liver disease induced by high-alcohol-producing Klebsiella pneumoniae
Source: J Gastroenterol. 2025 Dec 24;61(4):462–76. doi: 10.1007/s00535-025-02332-x (PMC13048965; doi:10.1007/s00535-025-02332-x)
Supplement: Supplementary file 1 — Supplementary file1 (DOCX 26524 KB) [file 535_2025_2332_MOESM1_ESM.docx]

**Supporting Information**

**
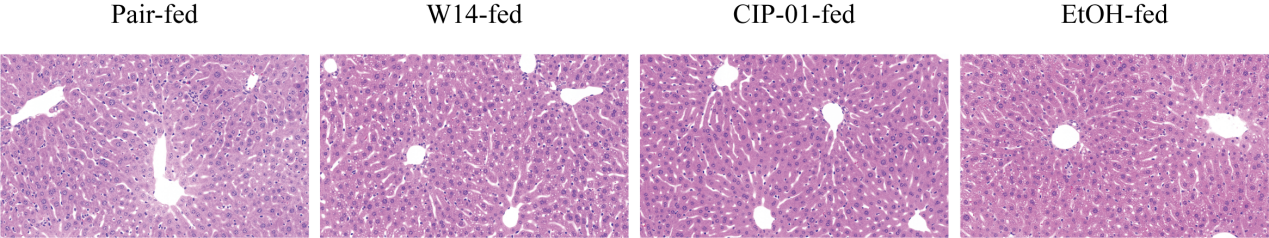
**

**Supplementary Figure 1 Analysis of hepatic damage.** *H&E* staining (20X) of liver sections from SPF mice fed with HiAlc *Kpn* W14, *B. bifidum* CIP-01, EtOH and a chow diet for 6 weeks.

**
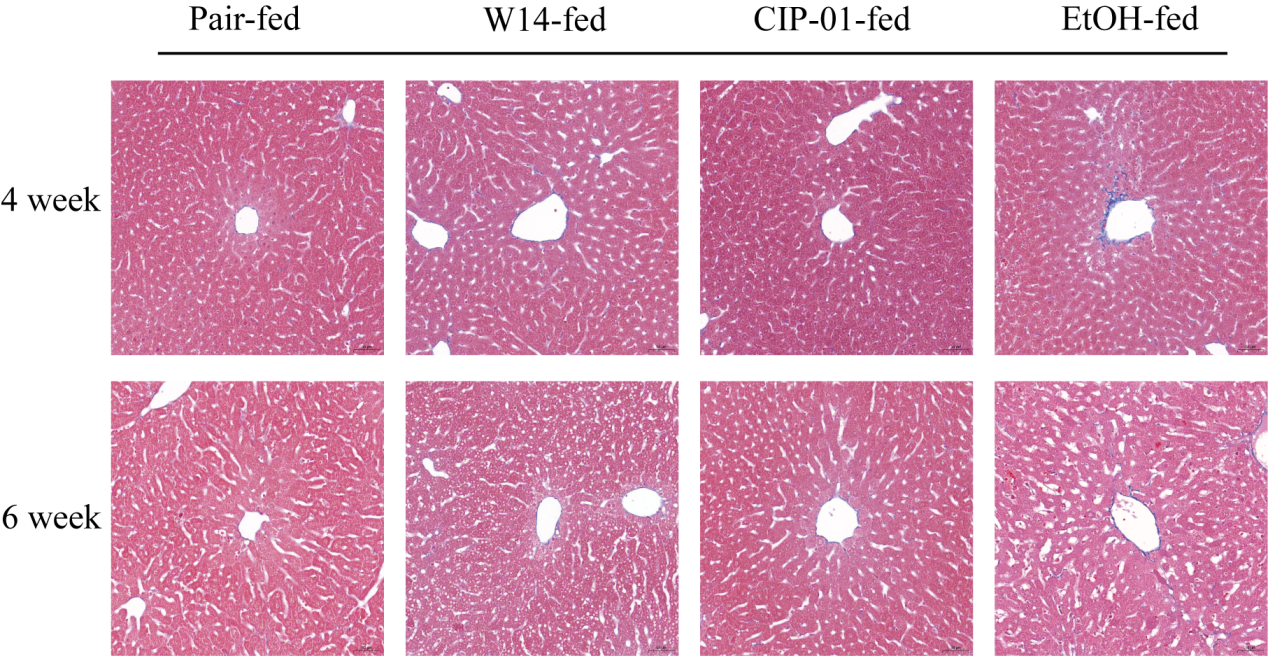
**

**Supplementary Figure 2 Analysis of hepatic fibrosis.** The deposition of collagen fibers in liver tissues was evaluated by masson staining.

**
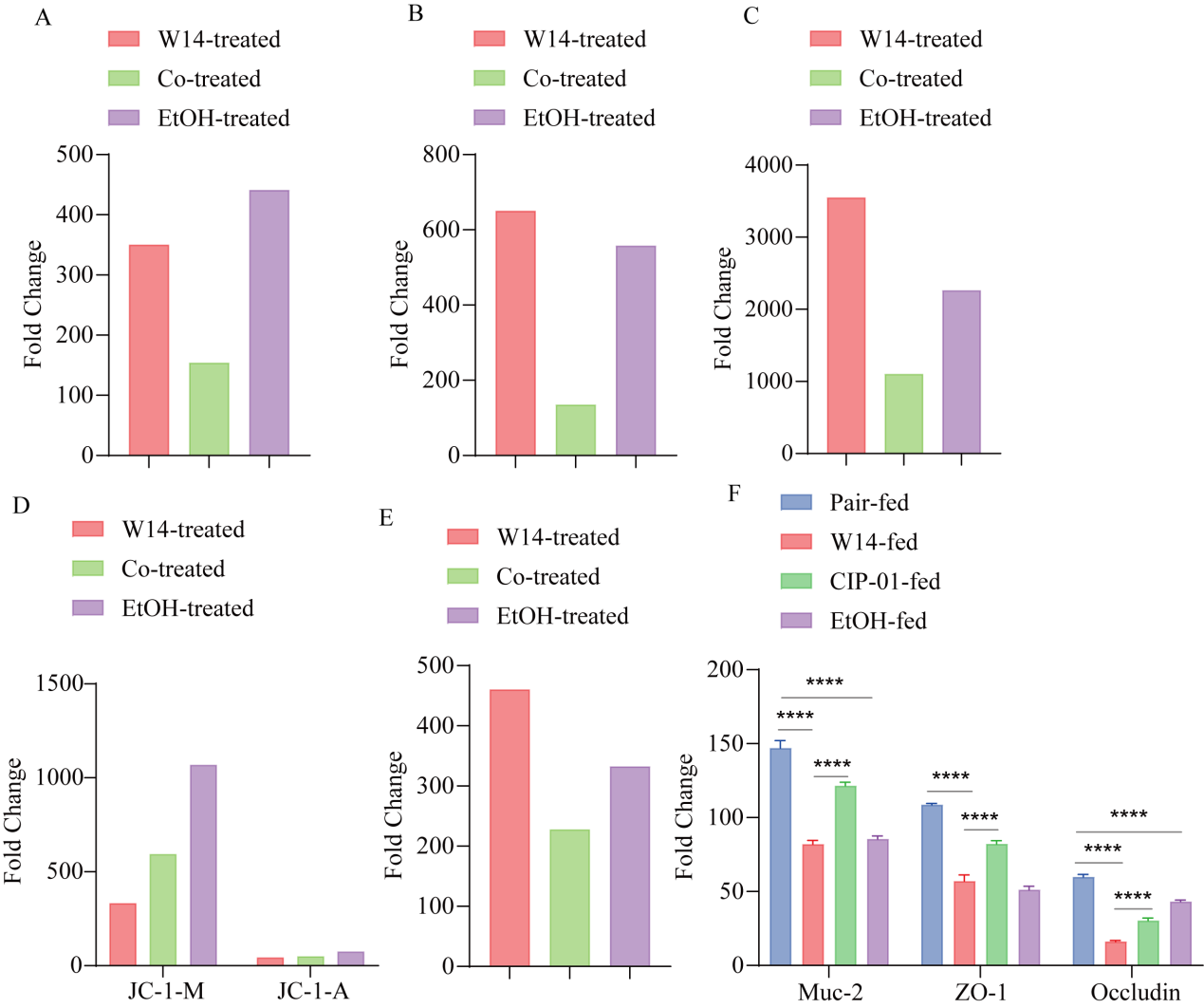
**

**Supplementary Figure 3 Quantification of fluorescence intensity in NCM460 cells.**

1. Fold changes of the fluorescence intensity of apoptotic signals (red).
2. Fold changes of the fluorescence intensity of ROS signals (red).
3. Fold changes of the fluorescence intensity of calcium influx (green).
4. Fold changes in mitochondrial membrane potential (ΔΨm).
5. Fold changes of the fluorescence intensity of ER signals (red).
6. Fold changes of ZO-1, Muc-2, and Occludin proteins in intestinal tissues in mice.

All data are presented as the mean ± SD. *ns,* *P > 0.05,* *^*^P < 0.05*, *^**^P < 0.01*, *^***^P < 0.005, ^****^P < 0.001*. *P* value was assessed by two - way ANOVA.

**
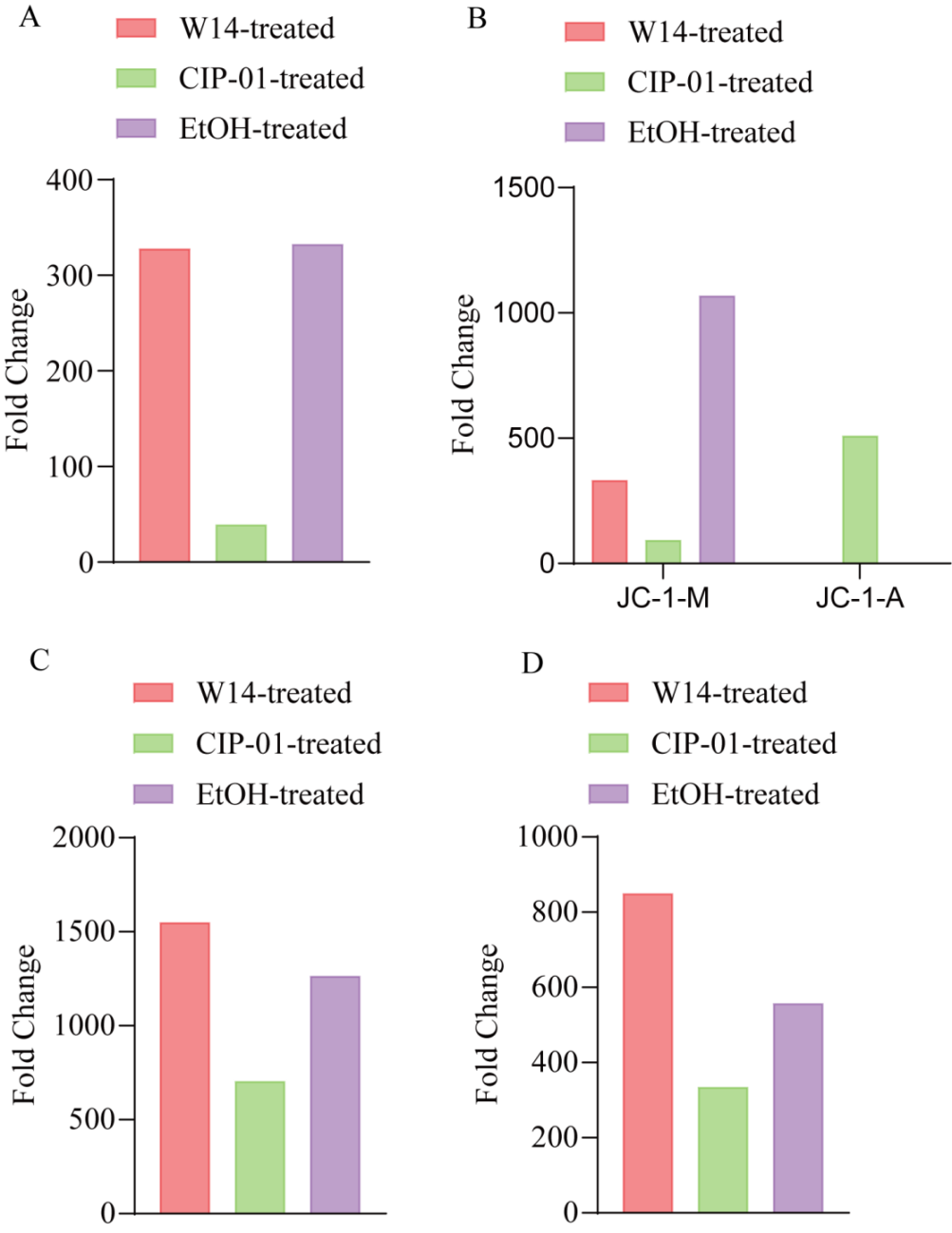
**

**Supplementary Figure 4 Quantification of fluorescence intensity in HepG2 cells .**

1. Fold changes of the fluorescence intensity of ER signals (red).
2. Fold changes in mitochondrial membrane potential (ΔΨm).
3. Fold changes of the fluorescence intensity of calcium influx (green).
4. Fold changes of the fluorescence intensity of ROS signals (red).

All data are presented as the mean ± SD. *ns,* *P > 0.05,* *^*^P < 0.05*, *^**^P < 0.01*, *^***^P < 0.005, ^****^P < 0.001*. *P* value was assessed by two - way ANOVA.


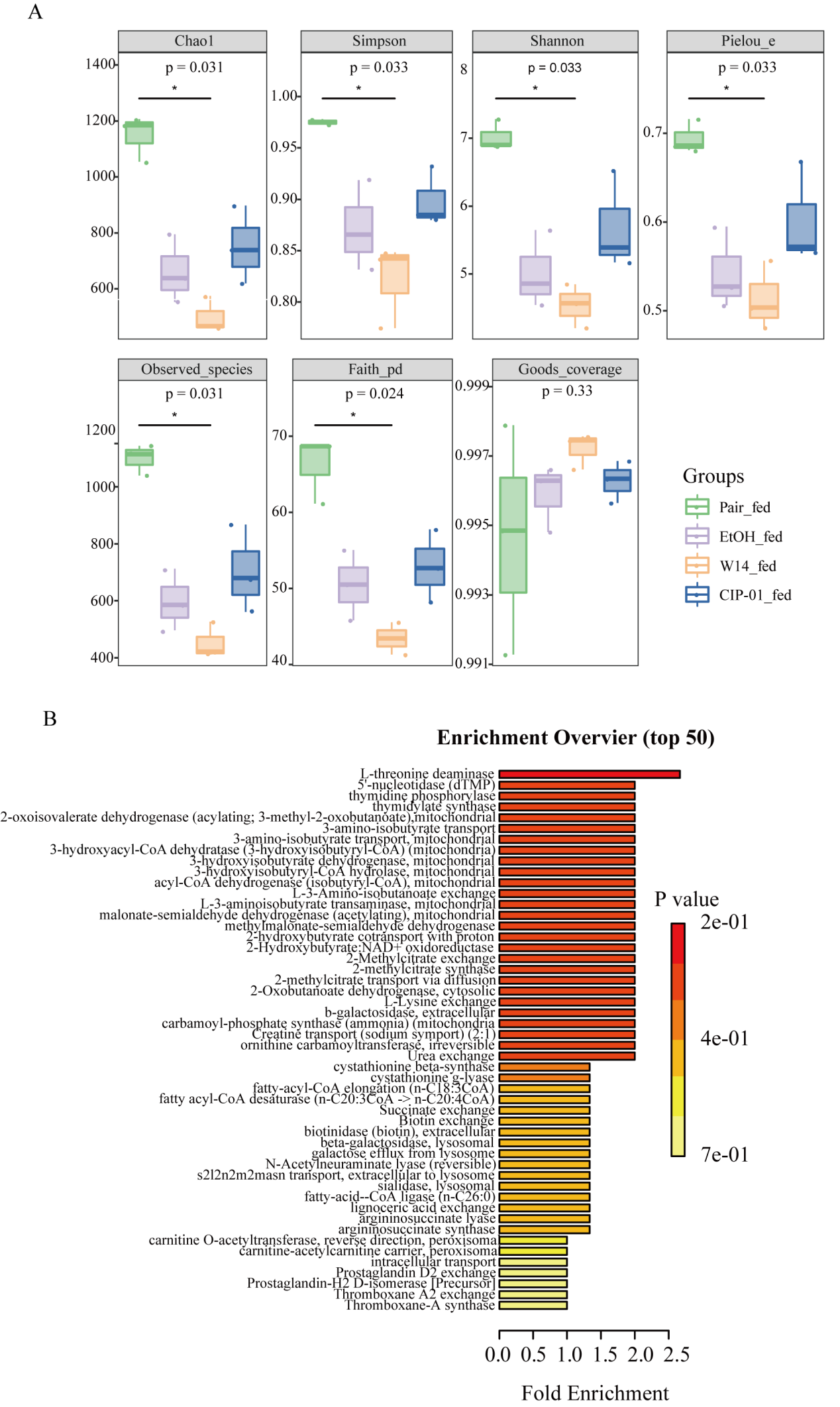


**Supplementary Figure 5 Multiple sequence analysis.**

1. Microbiota diversity analysis by 16S rRNA sequence in feces of experimental mice fed with *HiAlc Kpn* W14, *B. bifidum* CIP-01, EtOH and a chow diet for 4 and 6 weeks.
2. Enrichment analysis of differentially expressed metabolites in feces of experimental mice fed with *HiAlc Kpn* W14 and a chow diet for 4 and 6 weeks.


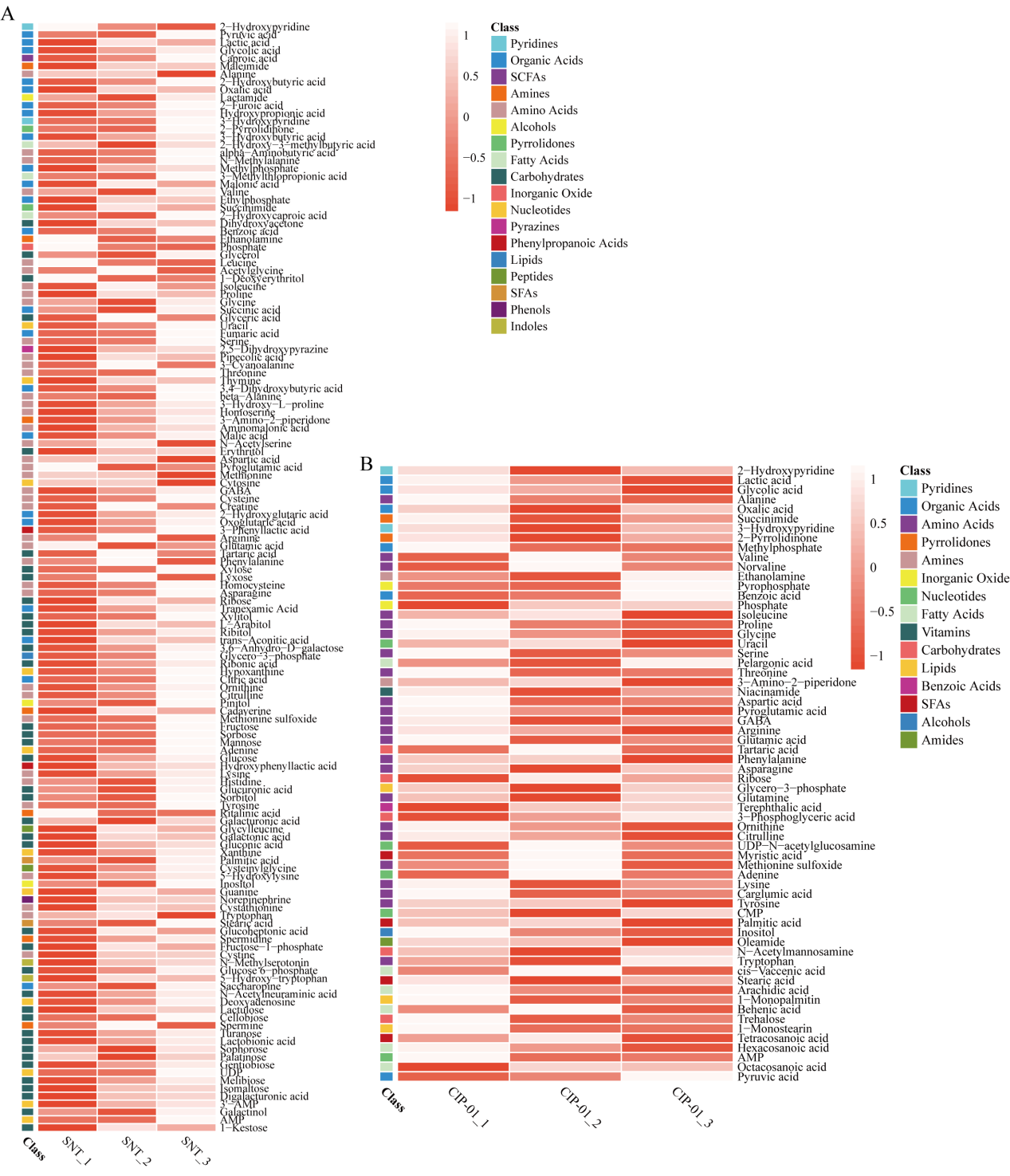


**Supplementary Figure 6 Metabolomics sequencing analysis of *B. bifidum* CIP-01.**

1. Heatmap showing the enriched metabolites in the culture medium supernatant of *B. bifidum* CIP-01.
2. Heatmap showing the enriched metabolites of *B. bifidum* CIP-01.

**
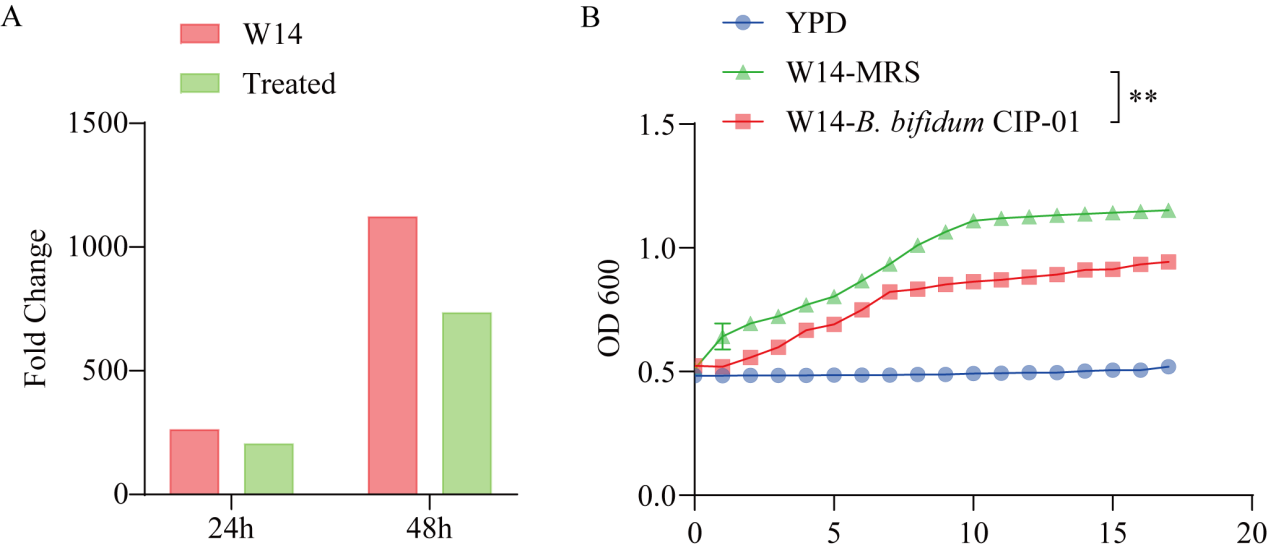
**

**Supplementary Figure 7**  ***B. bifidum* CIP-01 shows a negative effect on HiAlc *Kpn* W14.**

1. Quantitative analysis of the biofilm expression in 24 hours and 48hours.
2. Effect of *B. bifidum* CIP-01 strain on growth of HiAlc *Kpn* W14, MOI = 10, n = 3. All data are presented as the mean ± SD. *^**^P < 0.01*. *P* value was assessed by one - way ANOVA.

# Table S1. Primers for qPCR.

| Gene | Forward (5'-3') | Reverse (5'-3') |
| --- | --- | --- |
| Bax | GCTGCAGAAGGGTGGCAGGT | CTGTCACTGCGCGGGCCTGT |
| Bcl-2 | CAGACAAGAAGAGGTTGCC | CGTCAGTCAGTGTGTATG |
| CytoC | CAGACAAGAAGAGGTTGCC | CGTCATGGCAGTGTGTATTGG |
| Caspase3 | CAGACAAGAAGAGGTTGCC | CGTCAGGCAGTTTGTATTGG |
| Gapdh | CATGGCCTTCCGTGTTCCTA | CCTGCTTCACCACCTTCTTGAT |
